# Effects of mixing water and environmental pH value on the properties of sulfoaluminate cement-based ultra-high water materials

**DOI:** 10.1038/s41598-024-66618-5

**Published:** 2024-07-10

**Authors:** Yaohui Zhang, Chun Wang, Yumeng Ren, Zuqiang Xiong

**Affiliations:** 1 Department of Resources and Environment, Henan College of Industry & Information Technology, Jiaozuo, 454003 Henan China; 2 School of Energy Science and Engineering, Henan Polytechnic University, Jiaozuo, 454003 Henan China; 3 Sinosteel MaAnShan General Institute of Mining Research Co., LTD, Maanshan, 243000 Anhui China

**Keywords:** Engineering, Materials science

## Abstract

In order to grasp the influence of the pH value of mixing water and environmental water on the properties of ultra-high water materials, this article separately carried out the influence of different pH values of mixing water on the properties of ultra-high water materials and the conservation of high-water materials in water environments with different pH values. Using test methods such as loss of flow time, compressive strength, Scanning Electron Microscope (SEM), X-Ray Diffraction (XRD), Thermogravimetric-Differential Thermal Analysis (TG–DTA), to conduct regular exploration and mechanism analysis. The study found that with the continuous increase of the pH value of the mixing water, the loss of flow time of the ultra-high water material gradually decreased, and the compressive strength of the samples at the same age continued to increase. A lower pH value will affect the compressive strength of the consolidated body of the ultra-high water material, but when the pH = 13 in the reaction solution, the compressive strength of the consolidated body will no longer increase and begin to produce a weakening effect. The pH of the construction water for ultra-high water materials is recommended to be 4–13. At the same time, it was found that under the conservation of an acidic environment, the consolidated body was severely eroded and the strength loss was large. The acid–base environment of the goaf suitable for filling with the ultra-high water material should be between pH = 7–10 to ensure that the filling body is not weakened by erosion.

## Introduction

Ultra-high water filling materials (ultra-high water materials) were first proposed based on traditional high-water materials by Sun and Feng et al^1^. The material is composed of two components. The water-cement ratio can reach 6–11:1, and the volumetric water content is as high as 95%-97%. This material is mainly used for mine filling and mining, and the use of powder per unit volume is less, which greatly reduces cost ^1,2^. The main product of ultra-high water material is ettringite (AFt).

In mine filling and mining, mine water is generally used directly. The source of mine water is complicated, mainly including atmospheric water, surface water, underground water and underground wastewater. The mine water of different mines exhibits different acidity and alkalinity^3^. In particular, the problem of acidic mine water in China is particularly prominent. acidic mine water accounts for about 6.5%^4,5^. Acidic mine water generally refers to mine water with a pH value of less than 6, and most of it is between 2 and 4. In addition, when the formation water is alkaline and the sulfur content of coal seam is low, it will cause the mine water to behave alkaline^6^. Different pH values of mine water will not only affect the hydration reaction of ultra-high water materials, but also change the curing environment of ultra-high water materials to a certain extent.

At present, many literatures^7–11^ on ultra-high water materials mostly focused on their own properties, such as the preparation of the material and its setting and hardening properties, and the thermal stability of the main hydration products. The reaction product of ultra-high water material is mainly AFt. Numerous studies have carried out experiments on the influence of pH on the morphology and performance of AFt. The research results are different due to different experimental conditions and ways to obtain AFt^12,13^. It is found that the stable existence of AFt depends on the pH value of the liquid phase. The pH value range for the stable existence of AFt is 10.5–13.0^12,13^. Studies^14,15^ have shown that the morphology and size of AFt synthesized under different pH values are significantly different. Kishar et al^16^. used gypsum, lime and tricalcium aluminate (C_3_A) as raw materials to prepare AFt. The test results found that when the pH value in the liquid phase is lower than 10, no AFt is formed inside; and Gabrisova et al^17^. It is found that AFt is stable when the pH is greater than 10.7. At the same time, Zhang et al^18^. found that when the pH value in the liquid phase increases, the formation of AFt will be affected, and the conformation of AFt will decrease, changing from a columnar shape to a fine needle shape.

However, in different areas of the China, the pH of the mine water is different, especially the acidic environment will cause serious damage to the hardened body of the filling material. Scholars have done a lot of erosion experiments on cement, concrete materials, paste/high water filling materials and other materials in different acidic environments. Gao Meng^19–21^ studied the erosion test of water-rich material consolidation with a water-cement ratio of 3.5:1 in different acidic environments, chloride salts and sodium carbonate solution environments, using macroscopic performance testing, scanning electron microscope (SEM), X-ray powder diffraction (XRD) and Infrared spectroscopy and other research methods comprehensively analyze the degradation mechanism of water-rich materials in a corrosive environment. The research results show that the water-rich filling materials have a solidification effect on chloride ions, but the chloride salt solution has a corrosive effect on the water-rich filling materials. Sun Qi et al^22–25^. used three solutions of different concentrations of NaCl, Na_2_SO_4_ and MgSO_4_ to immerse the filling paste, and studied the influences of mine water corrosion on the strength of the filling paste. The research results show that the Na_2_SO_4_ solution affects the filling paste. The largest is the MgSO_4_ solution, followed by the NaCl solution. Liang et al^26^. accelerated the corrosion of concrete immersed in sodium sulfate and magnesium sulfate solutions through dry–wet cycle tests, and the results showed that the sulfate corrosion damage of concrete is a complex physical and chemical process. The corrosion damage mechanism of concrete in different sulfate solutions is different.

As a new type of filling material, the hydration process of ultra-high water material is carried out in a certain alkaline balance. In the reaction process of ultra-high water material, on the one hand, the pH value of the mixing water will directly change original pH value of the A and B slurry, after the two are mixed, further affects the pH value of the mixed slurry, and then affects the performance of the ultra-high water material; on the other hand, the ultra-high water material will be affected by the surrounding water environment during the hydration process. The pH value of the water environment will change the pH value of the ultra-high water material curing environment, and the different pH value of the curing environment will have a greater impact on the reaction products of the ultra-high water material. At the same time, the water-cement ratio of the ultra-high water material is as high as 6:1, its strength is low, and the corrosion resistance is poor. The pH value of the mixing water and the curing environment has a greater impact on its performance, but the above research content is currently rarely paid attention to by scholars. Therefore, it is necessary to carry out relevant experiments to explore the effect and mechanism of the pH value of the mixing water and the maintenance environment on the performance of ultra-high water materials. It can not only provide a basis for on-site construction, but also save water, and at the same time provide the possibility to utilize acid, alkaline and other industrial wastewater.

## Materials and methods

### Materials

The ultra-high water material is composed of two components, A and B. The A component is mainly made of sulphoaluminate cement clinker, and certain additives are added. The B component is made by mixing and grinding anhydrite and lime in a certain proportion, and add certain additives^27^. The chemical composition and mineral composition of sulphoaluminate cement clinker are shown in Tables 1 and 2. The specific surface area of anhydrite is 400 m^2^/kg, its chemical composition is shown in Table 3. The content of CaO in lime is 80%, and the specific surface area is 340m^2^/kg.

**Table 1:** Chemical composition of sulphoaluminate cement clinker/%.

CaO	Al_2_O_3_	Fe_2_O_3_	SO_3_	MgO	SiO_2_	Residue mass
45.16	29.53	3.83	9.39	0.81	8.61	0.41

**Table 2:** Mineral composition of sulphoaluminate cement clinker/%.

${\text{C}}_{4}{{\text{A}}}_{3}\overline{\text{S}}$	$\text{B-C}_{2}{\text{S}}$	${\text{C}}_{2}{\text{F}}$	$\text{f-S}{\text{O}}_{3}$
58.36	24.66	6.45	1.91

**Table 3:** Chemical composition of anhydrite/%.

SO_3_	SiO_2_	Al_2_O_3_	Fe_2_O_3_	CaO	MgO	Loss	∑
46.12	4.17	2.40	0.91	37.58	2.03	3.29	99.47

### Experimental program

#### The influence of water pH on the properties of ultra-high water materials

According to the pH value of mine water in different regions and industrial wastewater from different industries, the experiment designed five water pH values, namely 1, 4, 7, 10 and 13. The acidic water environment is diluted with sulfuric acid to a certain molar concentration, and the alkaline water environment is prepared with NaOH (analytical pure) powder. The water-cement ratio is 6:1, the water temperature is 20 °C, and the curing temperature is 20 °C.

In order to explore the effect of water with different pH values on the properties of ultra-high water materials, standard samples were prepared with water of different pH values. The samples were cured to 1d, 3d, 7d, 14d, 28d, and then tested for compressive strength, SEM, XRD diffraction, and TG–DTA, respectively, for mechanism exploration and analysis.

#### The influence of environmental water PH on the performance of ultra-high water material hardened body

**Figure 1:**
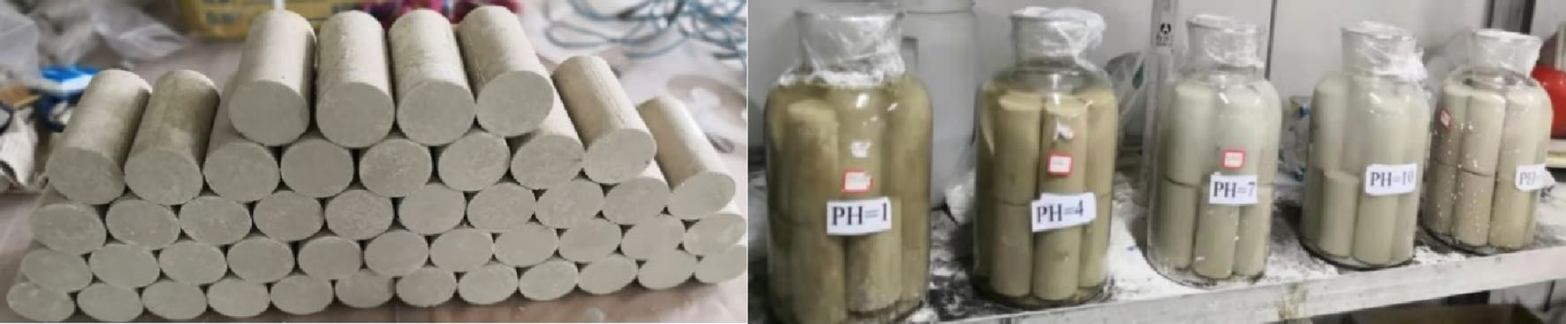
Samples preparation and erosion test process.

The sample is made of pure water with pH = 7, water-cement ratio is 6:1, and the reaction water temperature is 20℃. After pouring the sample, it is put into a curing box for curing. The curing age is 28 days and the curing temperature is 20℃. After the samples are cured to 28 days of age, they are put into 5L jars containing water with pH values of 1, 4, 7, 10 and 13, and soaked for 7d, 14d, 28d, 56d and 90d respectively. After being immersed to the test age, the compressive strength, SEM, XRD and Thermogravimetry–differential thermal analysis (TG–DTA) analysis were used to analyze the material properties and hydration products. The molded sample and the immersed sample are shown in Fig. 1.

### Methods

#### The loss of flow time

The test method of loss of flow time refers to the current ‘High Water Filling Material *MT/T420-1995* industry standard’.

#### Compressive strength tests

The compressive strength of cement paste (die diameter Φ50mm, high 100 mm) was tested by the same loading procedure as described in *GB/T17671-1999*. The tests were conducted at the age of 1d, 7d and 28d.

#### SEM

The piece samples after the termination of hydration and vacuum drying were used for SEM observation. Samples were gold coated and the observations were conducted under high vacuum condition.

#### XRD

The same powder sample was used for XRD. An X-ray diffractometer of Rigaku SmartLab was used for the measurements. The test voltage was 40 kV and the working current was 150 mA. The scanning was from 5° to 50° at a rate 10°/min.

#### TG–DTA

TG-DTA tests were conducted in a simultaneous thermal analyzer in N_2_ environment, and the heating temperature was from 20 to 900 ℃ by a heating rate of 10 ℃/min.

## The effect of mixing pH water on the properties of ultra-high water materials

### Analyses of setting time test

**Figure 2:**
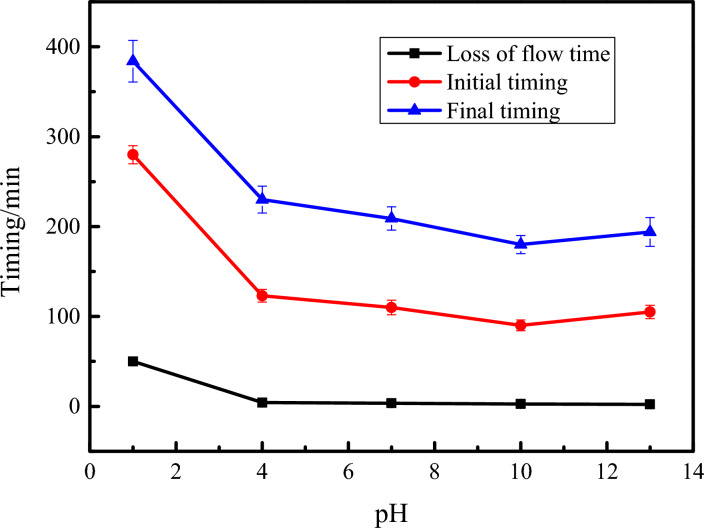
The effect of water pH on the loss of flow time of ultra-high water materials.

The fluidity loss time, initial setting time and final setting time of ultra-high water materials vary with the pH value of the water, as shown in Fig. 2. As the pH value of water increases, the time of losing fluidity, initial setting and final setting time of ultra-high water materials gradually decreases. When the pH value of the water is 1, 4, 7, 10 and 13, the corresponding the fluidity loss time is 50 min, 4.2 min, 3.5 min, 2.7 min and 2.2 min, and the initial setting time is 280 min, 123 min, 110 min, 90 min and 105 min, the final setting time is 384 min, 230 min, 209 min, 180 min and 194 min respectively. The B component of the ultra-high water material contains lime. In an acidic environment, the OH^−^ formed by its hydration will neutralize H^+^. When the pH of the water is in the range of 4–7, it was found that low acidity water had little effect on the flow time loss, initial setting and final setting time of the ultra-high water material. It may be that the neutralization reaction made the entire system alkaline. However, when the pH of the water is less than 4, it can be concluded that the fluidity loss time and the setting time of ultra-high water materials are greatly affected.

### Analysis of compressive strength test

**Figure 3:**
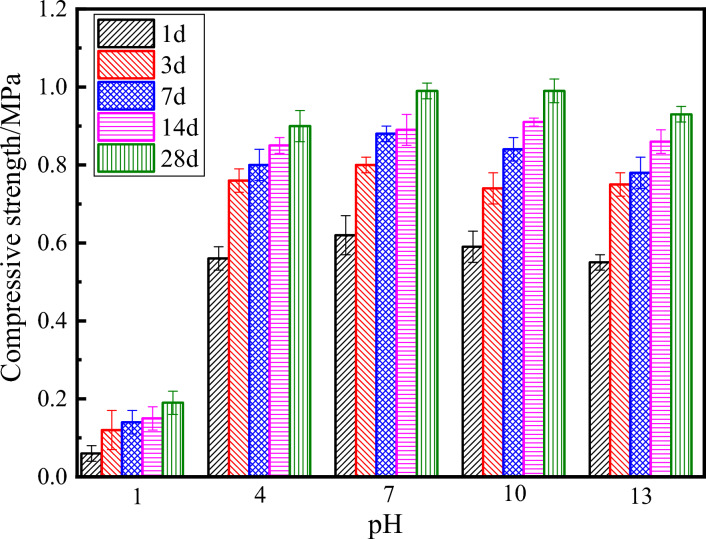
Compressive strength of high water materials at 1, 3, 7, 14 and 28d.

Figure 3 shows the compressive strength of the ultra-high water materials samples at 1, 3, 7, 14 and 28 days. Table 4 shows the reduction of compressive strength under other pH of water compared with the compressive strength of pH = 7.

**Table 4:** Reduction of compressive strength/%.

pH/Age	1	3	7	14	28
1	90.3	85	84.1	83.1	84.9
4	9.7	5	9.1	4.5	9.1
10	4.8	7.5	4.5	-2.2	0
13	11.3	6.3	11.4	3.4	6.1

The data is the reduction in the compressive strength of the hardened body mixed with other pH water compared with the ultra-high water material mixed with pH = 7 water.

With the increase of the pH value of water, the compressive strength of sample shows a trend of first increase and then decrease. When pH is 1, the compressive strength is the lowest. There is little difference between pH = 7 and pH = 10. When the pH increases to 13, the intensity decreases compared to pH = 7 and pH = 10. When the pH of water is 1, the compressive strength is the smallest, and the compressive strength of 28 days is 0.19 MPa, which is only 21% of pH = 4, which is consistent with the previous test results of the loss of flow time. However, the final strength of pH = 4, pH = 7, pH = 10 and pH = 13 is not much different, which indicates that a lower pH of water will affect the compressive strength of ultra-high water materials, and the increase of pH value is conducive to the formation of material strength, but When the pH of water increases to a certain level, its compressive strength no longer increases. Wu et al^28^. also found that with the increase of OH^−^ concentration, the later compressive strength of sulfoaluminate hardened body decreased instead. It may be that the OH^−^ concentration is too high, hydrated to form a large number of small-sized AFt surrounding the unhydrated particles, and a relatively dense hydration product layer is formed quickly, so that further hydration is inhibited^28^. This is consistent with the findings of this experiment. At the same time, with the increase of the curing age, it was found that the strength of each group of samples continued to increase.

### Analysis of SEM test

**Figure 4:**
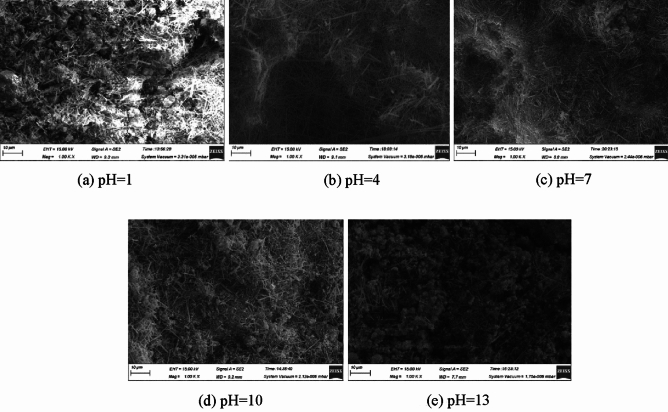
SEM of 1 day sample at different pH of water.

**Figure 5:**
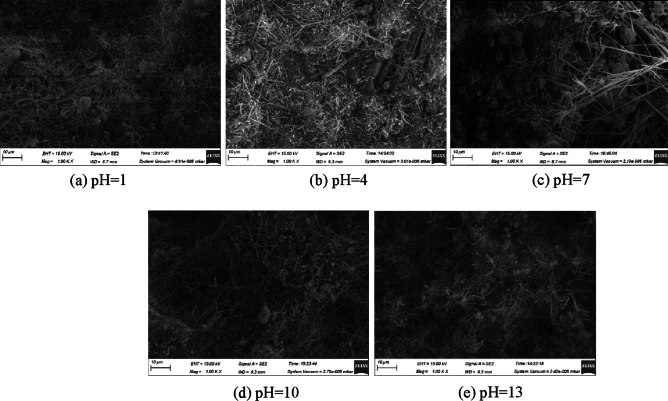
SEM of 7 days samples at different pH of water.

**Figure 6:**
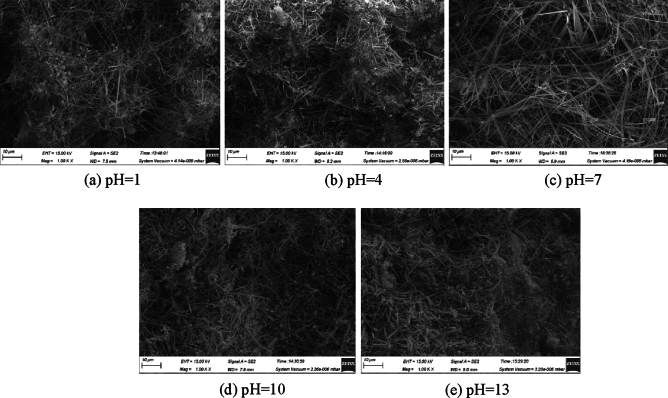
SEM of 28 days samples at different pH of water.

The SEM image of ultra-high water material with 1, 4, 7, 10 and 13 of water pH at age of 1, 7and 28 days are shown in Figs. 4, 5 and 6. The main hydration product of the ultra-high water material is AFt, which mainly exists in needle-like and short columnar forms. As the age increases, the content of AFt continues to increase, while the diameter and length of AFt are also increasing. This is consistent with the previous compressive strength test results. As the curing age of the sample increases, its compressive strength is also increasing.

At the same time, it is found that the pH of water will affect the morphology of hydration products of ultra-high water materials. At the age of 1 day, the sample with pH = 1 produces the least amount of AFt and the shortest diameter, most of which is extremely slender needle-like and sparsely distributed; the number and diameter of AFt in the sample with pH = 4 increase significantly. The distribution of AFt is relatively dense. As the pH value of water further increases, the content and compactness of AFt increase uniformly; when the pH value of water increases to 13, the amount of AFt formed on the surface of the sample is significantly reduced, and at the same time, the diameter and length of AFt are reduced. At the age of 7 days, the morphology of AFt inside the reaction solution sample at pH = 1 did not change much, but the density increased. The morphology of AFt inside the other pH samples all changed greatly, and AFt of larger size began to appear, and the density of AFt also increased. After reaching the age of 28 days, the internal morphology of the pH = 1 sample changed, and larger-sized AFt began to appear, which was roughly the same size as the AFt formed under the condition of pH = 4, but the overall density was lower than pH = 4 sample, AFt of pH = 7 sample has the best crossover, bonding ability, and the largest size, that is, its compressive strength is the highest; in particular, there is no large diameter in the pH = 10 sample AFt, pH = 13 does not appear in the sample with large diameter and long length, but its AFt distribution is relatively uniform, which also proves to a certain extent that too high alkalinity inhibits AFt. The shape of AFt changes the load-bearing capacity of the sample to a certain extent, but the effect is small, and the macroscopic compressive strength is not much different.

### Analysis of XRD test

**Figure 7:**
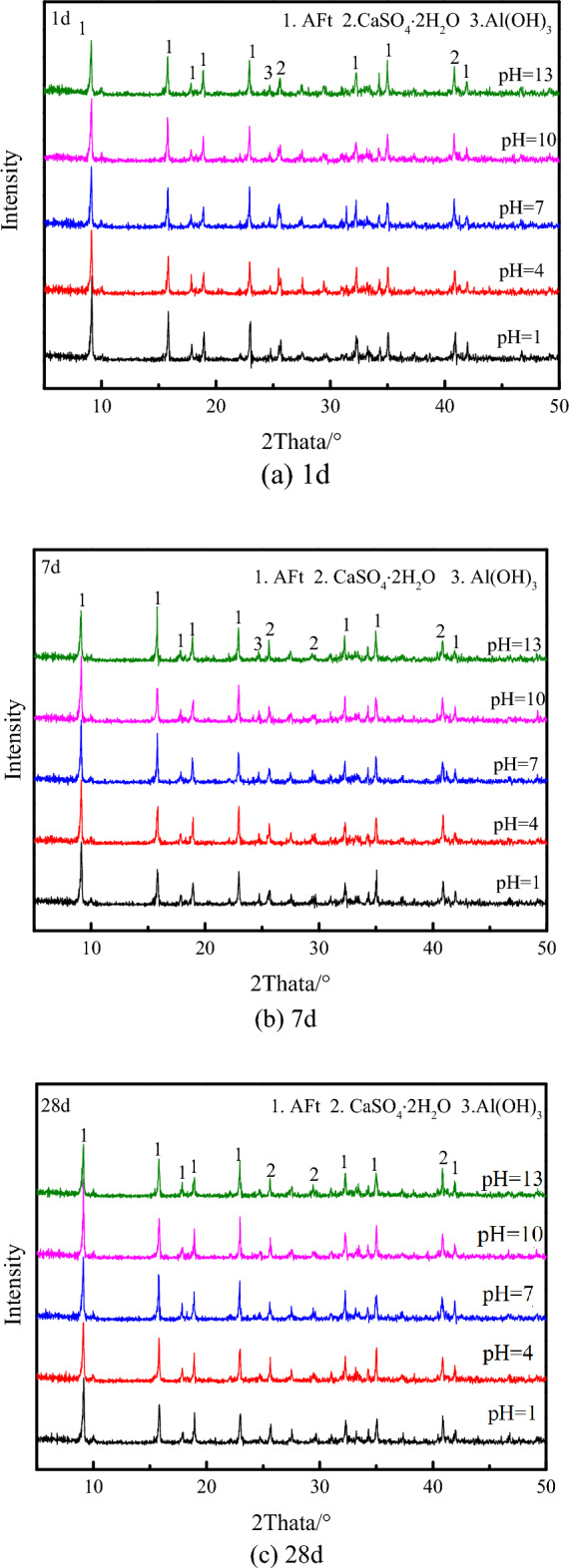
XRD analysis results of samples at each age under different acid–base reaction solutions.

Figure 7 shows the XRD of the ultra-high water materials samples at 1, 7 and 28 days. The pH of water has no effect on the type of hydration products of ultra-high water materials. The hydration products are mainly: AFt, CaSO_4_.2H_2_O and Al(OH)_3_. This is consistent with the test results of Zuo et al^29^. and Zhang et al^27^. Among them, at different pH, the number of AFt diffraction peaks at each age is the largest, and the diffraction intensity is the highest, indicating that AFt is still the main hydration product, and the ultra-high water material still produces a large amount of AFt in the acidic solution of pH = 1 and pH = 4, which is consistent with the results observed by SEM, that is, AFt is the main hydration product in each pH value sample at all ages.

### Analysis of TG-DTA test

**Figure 8:**
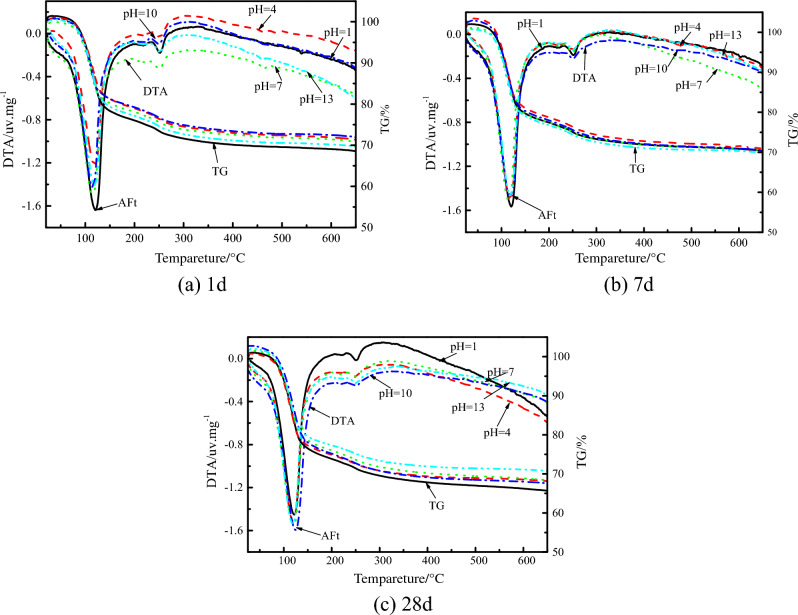
TG-DTA results of samples at different pH of water and different ages.

Figure 8 is a comparison chart of TG-DTA analysis curve results of the ultra-high water material samples at different water pH values and different ages. The AFt weight loss rate statistics are shown in Table 5. It can be seen there are mainly two endothermic peaks in the DTA curve of the above samples with different pH values for each age sample. The endothermic peak positions are 80–150℃ and 230–320℃. Comparing the spectrum of inorganic materials, the endothermic peak characteristics are consistent with AFt and Al(OH)_3_. The characteristics of the DTA curve indicate that a large amount of AFt is generated in the samples with different pH values of water in the ultra-high water material, which is consistent with the results of SEM and XRD analysis.

**Table 5:** Weight loss rate of AFt at different pH value of water.

Age/d	Weightlessness rate/%				
	pH = 1	pH = 4	pH = 7	pH = 10	pH = 13
1	24.63	25.12	26.10	25.50	27.07
28	27.12	28.45	28.64	29.75	27.90

Combining the weight loss rate of each pH value sample at different ages in the chart, it can be seen that the weight loss rate of AFt continues to increase with the extension of age, that is, the hydration process continues and the content of AFt continues to increase, which indicates that the pH value of water will not affect the progress of hydration of the ultra-high water material throughout the curing age. During the same age, as the pH value of water continues to increase, the weight loss rate of AFt increases continuously, indicating that the increase of pH value of water is beneficial to the formation of AFt, but when the pH value of water is too high, the weight loss rate of AFt is reduced, indicating that too high alkalinity will inhibit the formation of AFt. When the pH of the water is 4, 7 and 10, the final weight loss rate of AFt is not much different.

## The influence of pH value of environmental water on the properties of hardened body of ultra-high water material

### The influence of environmental water pH on the appearance of the ultra-high water materials

**Figure 9:**
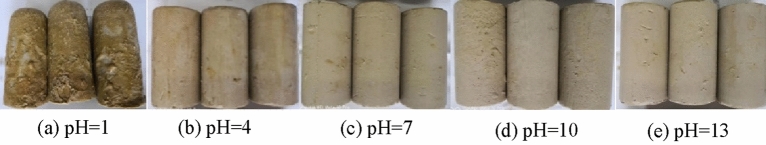
Samples at 7d age eroded by different water pH.

**Figure 10:**
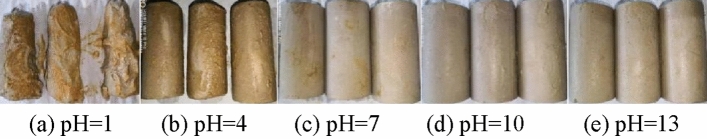
Samples at 14d age eroded by different water pH.

**Figure 11:**
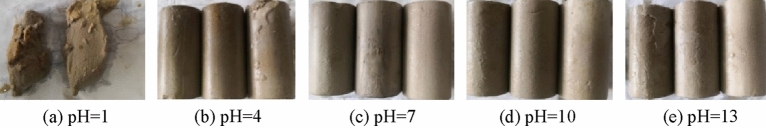
Samples at 28 d age eroded by different water pH.

**Figure 12:**
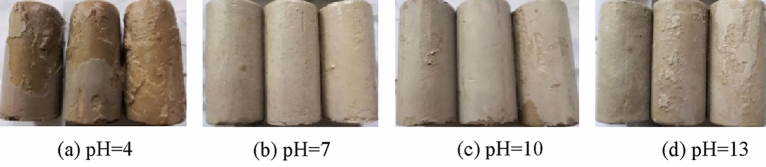
Samples at 56d age eroded by different water pH.

**Figure 13:**
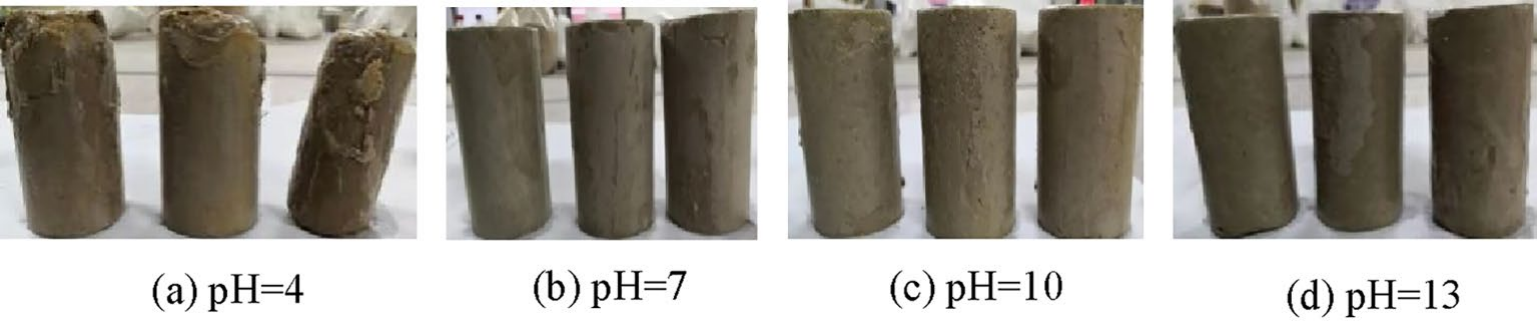
Samples at 90d age eroded by different water pH.

Figures 9, 10, 11, 12 and 13 shows the appearance of the ultra-high water material sample of different ages in different acid–base environments after being eroded. It can be seen that after immersing in a water environment with pH = 1 for 7 days, the surface of the sample is obviously corroded, the color of the sample surface becomes brown, and the size is reduced. When the immersion age was extended to 28d, the cylindrical specimens were completely eroded. After being immersed in a water environment with pH = 4 for 7 days, only the color of the sample changed, but the size of the sample did not change significantly. When the sample is immersed for 90 days, the thickness of the soft layer of the sample is further increased, the size of the sample is significantly changed, and the appearance of the sample shows a significant erosion effect. The samples were immersed in a water environment with pH = 7, pH = 10 and pH = 13 for different ages, and the size of the samples did not change significantly.

Thus, the acidic environment will corrode ultra-high water materials, and as the pH value decreases, the corrosion will become more serious. Neutral and alkaline environments have basically no effect on the appearance of ultra-high water material samples.

### The influence of environmental water pH on the compressive strength of the sample ultra-high water materials

**Figure 14:**
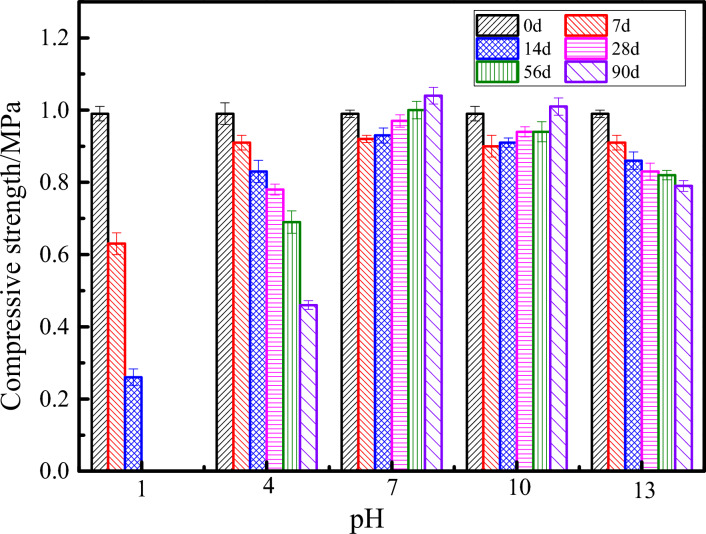
Compressive strength at different pH erosion environments.

Figure 14 shows the compressive strength of ultra-high water materials samples in different pH erosion environments at 0, 7, 14, 28, 56 and 90 days. When the pH of corrosive environment water is 1, 4 and 13, as the curing age increases, it is obvious that the compressive strength of the sample gradually decreases. When the pH of curing environment is 1, the sample has no compressive strength after 14 days of curing. After the samples were soaked in pH = 4 and 13 environments for 90 days, the compressive strength lost 53.5% and 20.2%, respectively, which greatly reduced the load-bearing capacity. On the contrary, when the pH of corrosive environment water is 7 and 10, as the curing age increases, the compressive strength of the sample first decreases and then increases. When the samples were soaked in pH = 7 and 10 environments for 90 days, the compressive strength increased 5.1% and 2%, respectively.

Compared with the environment at pH 7, the compressive strength reduction of samples under other conditions is shown in Table 6. It can be concluded that the low-alkali environment water has little effect on the compressive strength of the ultra-high water material hardened body, followed by the low acid and strong alkali environment, and the strong acid environment has the greatest influence on the strength of the ultra-high water hardened body.

**Table 6:** Reduction of compressive strength/%.

pH/Age	7	14	28	56	90
1	31.52	72.04	100	100	100
4	1.09	10.75	19.59	31.00	55.77
10	2.17	2.15	3.09	6.00	2.88
13	1.09	7.53	14.43	18.00	24.04

The data is the reduction in compressive strength under other pH conditions compared to the hardened body of high-water material immersed in environmental water at pH = 7.

### Analysis of SEM test results

**Figure 15:**
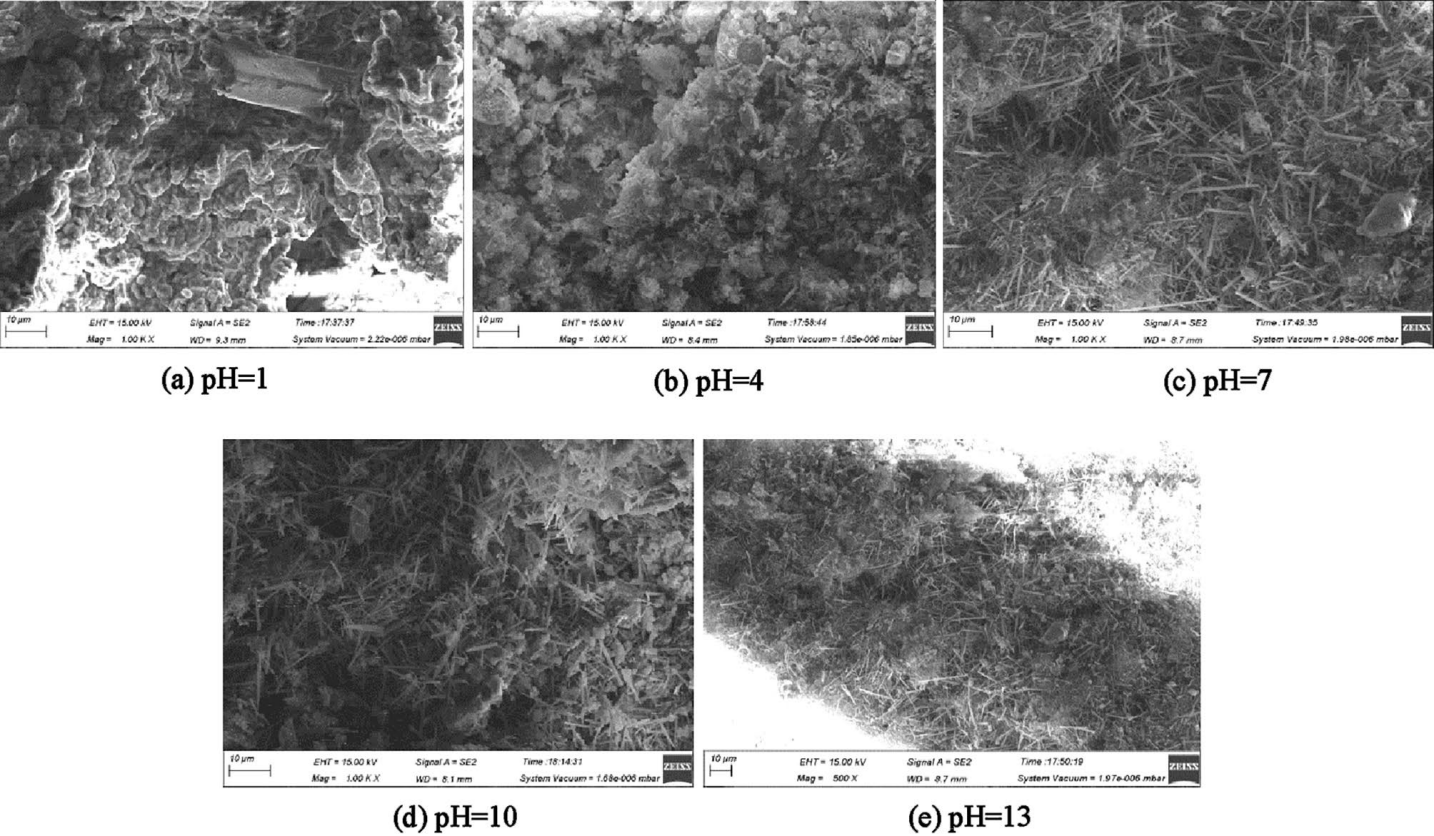
Soaking for 90 d with pH = 13 sodium hydroxide solution.

Figure 15 shows the SEM of ultra-high water materials samples in different pH erosion environments at age 90 days. It can be seen from Fig. 15a that after 90 days of erosion in the pH = 1 solution, the internal morphology has undergone tremendous changes from the 28d age microscopic morphology under standard curing conditions. You can see that there are strong prisms distributed. There are more clusters around the pillars, and there are almost no AFt crystals. When pH = 4, the sample contains a very small amount of fine and short AFt.

It can be seen from the following Fig. 15c–e that after 90 days of soaking in a pH = 7–13 solution environment, there is a large amount of AFt in the sample. Different from the standard curing conditions, the morphology of AFt has changed, that is, the AFt has changed from a large needle thread and columnar AFt to densely packed, intersecting short needles and columnar AFt. The content of AFt is not reduced and the density is increased. As the pH value increases, after immersing in a solution with a pH of 13 for 90 days, the AFt is wrapped by more flocs and jelly, and the gaps between the AFt become larger, and the content is lower than pH = 7 And the pH = 10 environment will reduce the load-bearing capacity of the hardened body, which is consistent with the previous compressive strength test results.

### Analysis of XRD test results

**Figure 16:**
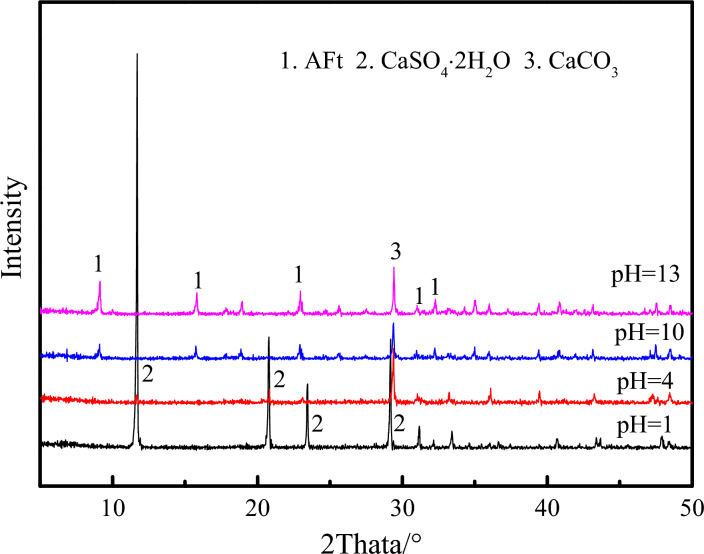
XRD of 90-day samples corroded by different pH water environment.

Figure 16 shows the XRD of the hardened body of ultra-high water material after being corroded by different pH corrosive environments for 90 days. The main hydration products in XRD analysis are AFt, dihydrate gypsum and calcium carbonate. When the pH of water is found to be 1 and 4, the diffraction peak of AFt, the main hydration product of the hardened ultra-high water material, disappears, and the diffraction peak of gypsum is detected. This is mainly because AFt ionizes Ca^2+^ and SO_4_^2−^ in an acidic environment (See Eq. 1).

1 $${\text{C}}_{{3}} {\text{A}}\cdot{\text{3CaSO}}_{{4}} \cdot{\text{32H}}_{{2}} {\text{O }} \to {\text{6Ca}}^{{{2} + }} + {\text{ 2Al}}\left( {{\text{OH}}} \right)_{{4}}^{ - } + {\text{3SO}}_{{4}}^{{{2} - }} + {\text{4OH}}^{ - } + {\text{26H}}_{{2}} {\text{O}}$$

When the hardened body of ultra-high water material is immersed in an acidic environment prepared by sulfuric acid, it will suffer double corrosion, first of all, the H^+^ in the acidic environment will neutralize with the OH^-^, then the free calcium ions inside the specimen will increase, which will react with SO_4_^2−^ as Eq. 2.

2 $${\text{Ca}}^{{{2} + }} + {\text{SO}}_{{4}}^{{{2} - }} + {\text{2H}}_{{2}} {\text{O}} \to {\text{CaSO}}_{{4}} \cdot{\text{2H}}_{{2}} {\text{O}}$$

The reaction causes the pore size of the hardened body to further increase, which is conducive to the further erosion of H^+^ and SO_4_^2−^, and finally changes the mineral composition and crystal structure of the material, resulting in a change in the volume of the material, and ultimately damage^30^.

When pH = 10 and pH = 13, the main product on the hardened body surface is still AFt. The result is consistent with the research results of Ma et al., AFt has inconsistent dissolution characteristics in different alkaline solutions, and OH^-^ has an inhibitory effect on the dissolution ability of Al(OH)_4_^-^, Ca^2+^ and SO_4_^2−^ in AFt^31^. This shows that the ultra-high water material has a greater impact on AFt in an acid environment. The decomposition of AFt changes the microstructure of the ultra-high water material hardened body, thereby reducing the compressive strength of the hardened body. This is basically consistent with the results obtained by the compressive strength test and SEM.

### Analysis of TG-DTA test results

**Figure 17:**
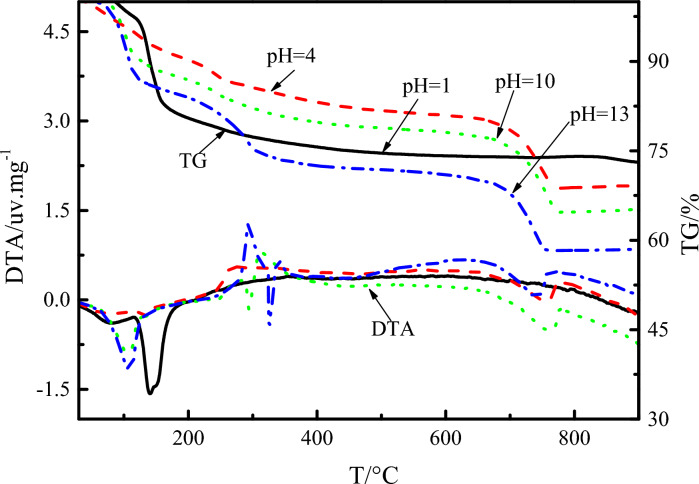
TG-DTA date of samples after soaking in different pH environment for 90 days.

Figure 17 shows the TG-DTA of ultra-high water materials samples in different pH erosion environments at age 90 days. After the test sample was immersed in a solution of pH = 1 for 90 days, the DTA curve showed an obvious endothermic peak at about 142℃. The endothermic peak is the characteristic curve of the endothermic peak of gypsum. The gypsum absorbs in the range of 120–200 ℃. Heat is accompanied by weight loss and eventually becomes anhydrous gypsum. After the sample was immersed in a solution of pH = 10 and pH = 13 for 90 days, the DTA curve showed three main endothermic peak intervals of 90–120 ℃, 290–340 ℃ and 680–770 ℃, compared with inorganic materials thermal analysis chart curve, the three main endothermic peak intervals are the characteristic curves of AFt, Al(OH)_3_ and CaCO_3_. The presence of calcium carbonate may be the carbonization of the sample, or the admixture from the ultra-high water material. In the range of 290–340 °C, Al(OH)_3_ partially loses OH^-^ after it absorbs heat. After heating continues, the endothermic heat is accompanied by weight loss, and most of the OH^-^ is lost and converted into diaspore. It is also consistent with the results obtained by XRD analysis, and it can be inferred that the gel-like substance observed in the SEM is Al(OH)_3_.

It can be found from the TG curve and Table 7 that when the pH of the corroded water is 10, the most AFt is formed in the hardened body of the ultra-high water material, and the strength of the ultra-high water material is mainly derived from AFt, which is consistent with the compressive strength test results.

**Table 7:** TG of hardened body hydration products after 90 days of erosion in different pH environments.

pH	Weightlessness rate/%			
	C_3_A·3CaSO_4_·32H_2_O	CaSO_4_·2H_2_O	CaCO_3_	Al(OH)_3_
1	2.42	17.13	0	0
4	5.10	4.13	11.36	0
10	13.48	0	12.33	9.47
13	12.20	0	12.50	10.60

## Discussions

The effects of the pH of the water and the pH of the curing environment on the properties of ultra-high water materials have been experimentally studied. The macroscopic use of the loss of flow time and compressive strength tests, the microscopic use of SEM, XRD and TG-DTA to observe the morphology, type and content of hydration products, meanwhile, to explore the mechanism.

1. Experiments have found that the pH of water has a significant impact on the properties of ultra-high water materials. With the continuous increase of the pH value of the water, the loss of flow time of the ultra-high water material gradually decreases, and the compressive strength of the samples at the same age increases. When the pH = 1, the material bleeding is serious, and the loss of flow time is as long as 50 min. The compressive strength of 28d age is only about 21% of that at pH = 4, while pH = 4, pH = 7, pH = 10 and pH = 13, there is little difference in the final strength. The pH of water does not affect the types of hydration products of ultra-high water materials, but at lower pH it will affect the length and diameter of the AFt, thereby affecting the compressive strength. But when the pH of the water is 13, the compressive strength of the sample no longer increases, and the weakening effect begins. The suitable pH of the construction water for ultra-high water materials is 4–13.
2. After the ultra-high water material hardened body is placed in an acidic water environment, the appearance of the ultra-high water material changes significantly. The stronger the acidity, the more severe the corrosion of ultra-high water materials and the more obvious the loss of compressive strength. Experiments have found that in an acidic environment, the presence of AFt is very small. The main hydration product is dihydrate gypsum. The compressive strength of ultra-high water materials mainly comes from AFt, and its dissolution reduces the compressive strength of ultra-high water materials. In a water environment with a pH value greater than 7, the appearance of ultra-high water materials is less corroded. After the soaking time reaches 90 days, the compressive strength increases, but as the pH value increases to 13, the compressive strength gradually weakens. The surrounding water environment of the mining area suitable for filling with ultra-high moisture materials should be between pH = 7–10 to ensure that the filling materials are not weakened by erosion.

## Conclusions

By exploring the effects of mixing water and environmental pH value on the properties of sulfoaluminate cement-based ultra-high water materials, the following conclusions can be drawn:

1. A lower pH value will affect the compressive strength of the consolidated body of the ultra-high water material, but when the pH = 13 in the reaction solution, the compressive strength of the consolidated body will no longer increase and begin to produce a weakening effect. The pH of the construction water for ultra-high water materials is recommended to be 4–13.
2. Under the conservation of an acidic environment, the consolidated body was severely eroded and the strength loss was large.
3. The acid–base environment of the goaf suitable for filling with the ultra-high water material should be between pH = 7–10 to ensure that the filling body is not weakened by erosion.

## Data Availability

The data used to support the findings of this study are included within the article.
